# Supplementary material for: A normative modelling approach reveals age-atypical cortical thickness in a subgroup of males with autism spectrum disorder
Source: Commun Biol. 2020 Sep 4;3:486. doi: 10.1038/s42003-020-01212-9 (PMC7474067; doi:10.1038/s42003-020-01212-9)
Supplement: Supplementary file 1 — Supplementary Information [file 42003_2020_1212_MOESM1_ESM.pdf]

__Supplementary information for:__ A normative modeling approach reveals age-atypical cortical thickness in a subgroup of males with autism spectrum disorder

Table of contents

SITE AND PARTICIPANT INFORMATION 2

Supplemental Figure S1: Site distribution 2

QUALITY CONTROL 2

Supplemental Figure S2: Euler quality control 4

SENSITIVITY ANALYSIS 4

Supplemental Figure S3: Sensitivity analyses 7

BOOTSTRAPPING 7

Supplemental Figure S4: bootstrap validation 8

COMPARISON TO CENTILE MODELLING OF NORMATIVE DEVIATION 8

Supplemental Figure S5: Centile vs LOESS regression 9

INDIVIDUAL SUMMARY RATIOS 9

Supplemental Figure S6: Global individual W-Score ratios 10

Supplemental Figure S7: Outlier age distribution per brain region 11

AGE-RELATED CT DEVIANCE RELATIONSHIPS WITH SRS AND ADOS 12

Supplemental Figure S8: Phenotype – W-Score correlations 12

SURFACE AREA, LGI AND VOLUME 13

Supplemental Figure S9: Variance contribution across measures 13

Volume *13*

Supplemental Figure S10: Canonical case-control comparisons for cortical volume 13

LGI *14*

Supplemental Figure S11: Canonical case-control comparisons for local gyrification 14

Area *15*

Supplemental Figure S12: Canonical case-control comparisons for surface area 15

MULTIVARIATE CLUSTERING 15

Supplemental Figure S13: tSNE Clustering 16

REFERENCES 17

Site and participant information

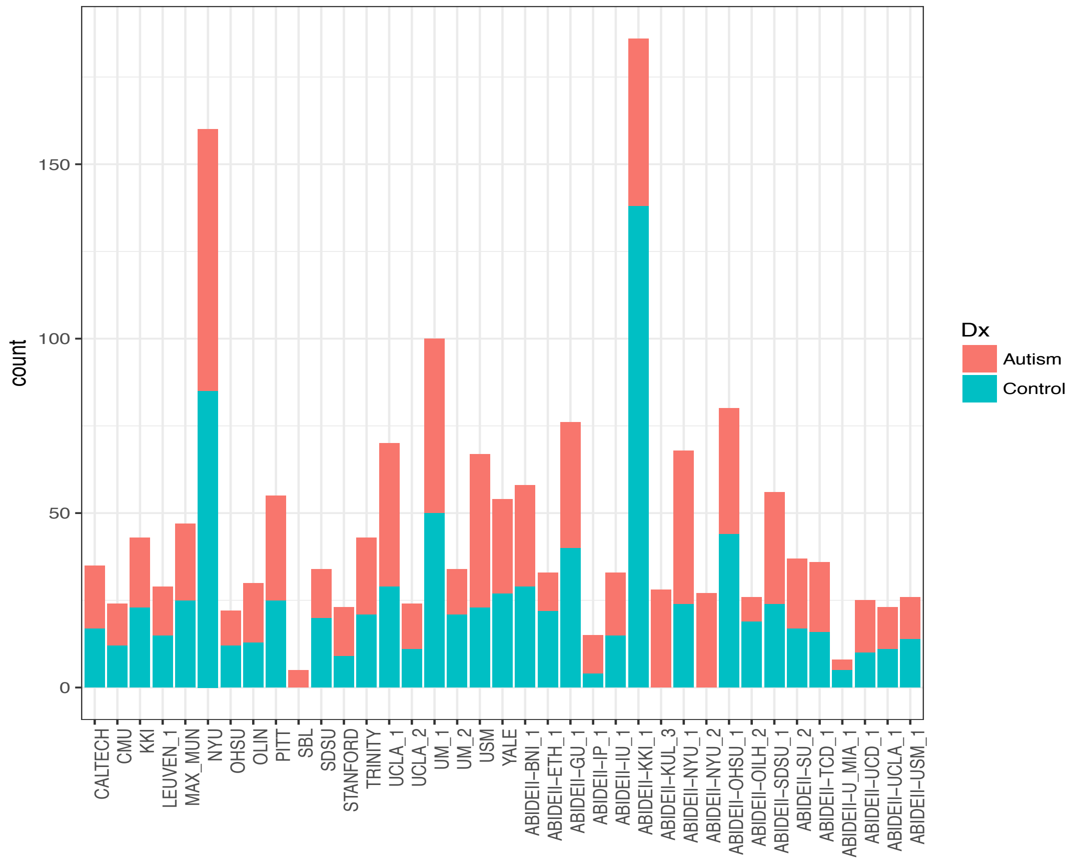

***Supplemental Figure 1:** Site distribution*

Quality control

To assess quality of Freesurfer reconstructions we computed the Euler index (Rosen et al., 2018). The Euler number is a quantitative index of segmentation quality and has shown high overlap with manual quality control labelling. In the full sample we found a small but significant difference in both hemispheres (Figure S2) with the autism group having overall worse scan quality (d = 0.176 and d = 0.187 for left and right hemisphere respectively). We excluded all subjects with a Euler score of 300 or higher in either hemisphere. After thresholding based on Euler indices and after re-running the sample consisted of 870 individuals with autism and 870 neurotypical individual, matched on Age, Euler and IQ.

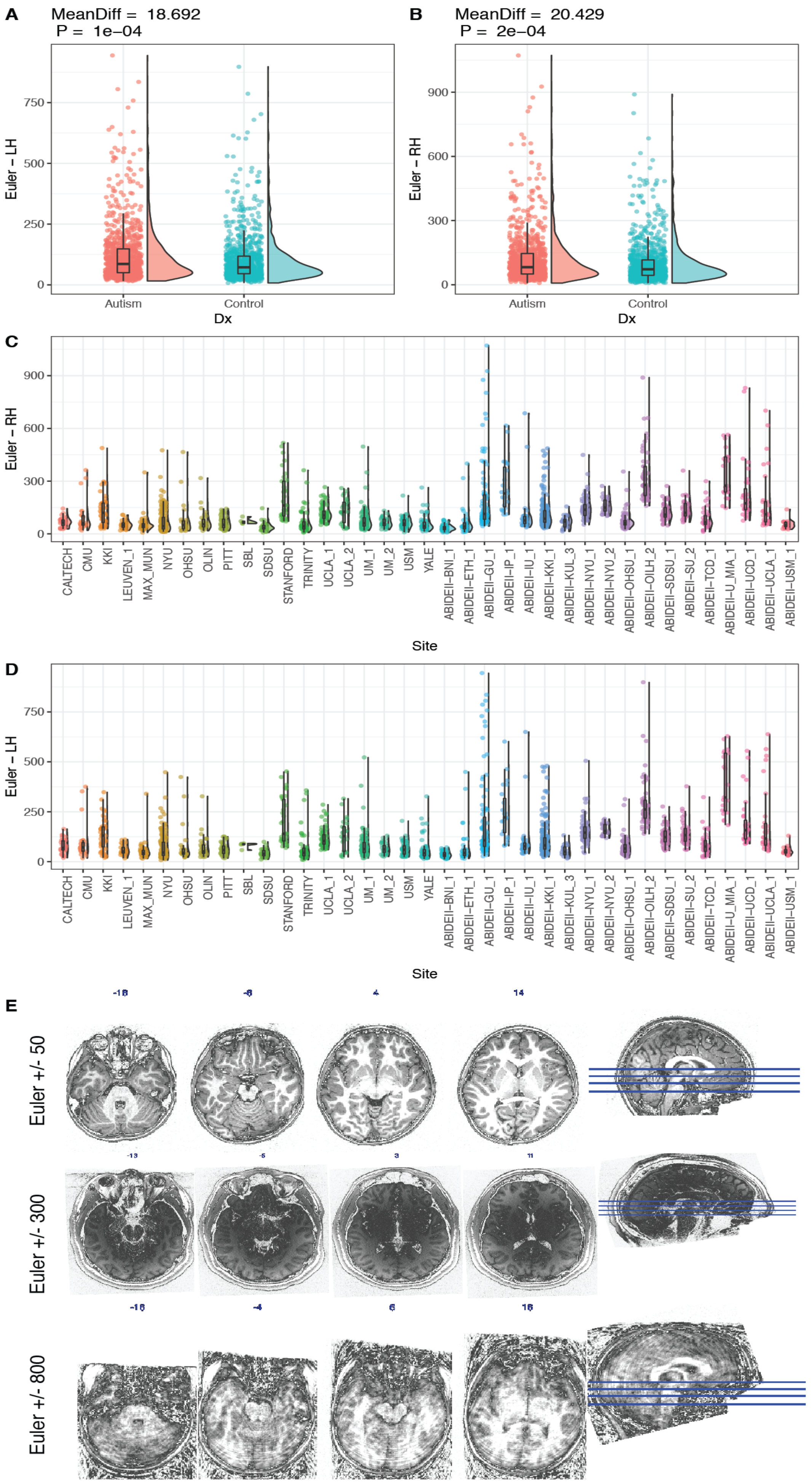
A
MeanDiff = 18.692
P = 1e−04
Euler – LH
Autism
Control
Dx
B
MeanDiff = 20.429
P = 2e−04
Euler – RH
Autism
Control
Dx
C
Euler – RH
CALTECH
CMU
KKI
LEUVEN_1
MAX_MUN
NYU
OHSU
OLIN
PITT
SBL
SDSU
STANFORD
TRINITY
UCLA_1
UCLA_2
UM_1
UM_2
USM
YALE
ABIDEII−BNI_1
ABIDEII−ETH_1
ABIDEII−GU_1
ABIDEII−IP_1
ABIDEII−IU_1
ABIDEII−KKI_1
ABIDEII−KUL_3
ABIDEII−NYU_1
ABIDEII−NYU_2
ABIDEII−OHSU_1
ABIDEII−OILH_2
ABIDEII−SDSU_1
ABIDEII−SU_2
ABIDEII−TCD_1
ABIDEII−U_MIA_1
ABIDEII−UCD_1
ABIDEII−UCLA_1
ABIDEII−USM_1
Site
D
Euler – LH
Site
E
Euler +/- 50
Euler +/- 300
Euler +/- 800

Supplemental Figure 2: Euler quality control

Panels A and B list the Euler indices for both left and right hemisphere, p-values for group differences were established with two-sided permutation testing (20000 permutations), Cohen's d was computed using custom R code https://github.com/mvlombardo/utils/blob/master/cohens_d.R. Panels C and D show the left and right hemisphere Euler distribution across the different sites included in ABIDE. Panel E shows 3 example scans for Euler indices of 50, 300 and 800 respectively. Raincloud plots were created using: https://micahallen.org/2018/03/15/introducing-raincloud-plots/

Sensitivity Analysis

To further assess the potential impact of data quality in the present study we processed the resting-state fMRI data to obtain estimates of in-scanner head-motion in the form of framewise displacement. We found that similar to the Euler index there were systematic group differences in in-scanner head motion (Figure S3.A), we also find that there were small significant correlations between in-scanner absolute, negative and positive w-score ratios ($r = 0.15$, $r = 0.16$ and $r = 0.09$ respectively, all $p < .05$, Figure S3.B). Then we assessed whether the extracted w-scores were spatially correlated with either Euler or head motion and found small (mostly negative) correlations ranging from $r = -0.18$ to $r = 0.14$ (Figure S3.C). Thus, we subsequently included both Euler index and framewise displacement as confound variables in all models.

Then, to systematically evaluate whether either motion or reconstruction quality impacted any of our outcome measures we conducted a cross-validation analysis by systematically excluding the top 5% of motion subject and top 5% of Euler subjects and assessed the spatial correlation in resulting Cohen's D maps (Figure S3.D). Resulting maps were highly consistent, with the lowest correlation ($r = 0.7$) between the sample with 95% and 75% of Euler subjects included, which is reflective of a decrease in sample size. We also assess the effect on the resulting one-sample assessment of the w-score between a model including motion as a confound and a model not including the motion confound (Figure S3.E). This showed near perfect consistency in spatial topology ($r = 1.00$, $p < .0001$). In similar fashion we assessed the case-control differences in a model with and without motion (Figure S3.F) and again found high consistency in spatial topology ($r = 0.96$, $p < .0001$).

To more specifically assess the relation between w-score ratios and head-motion we visualised their individuals correlations and observed that despite the small correlations there was little indication that high w-score ratio individuals (ratio > .5) were also the individuals with high

motion (Figure S3G-I). To systematically assess their influence, we removed the top 5% of motion autism individuals. We then reassessed the correlation (Figure S3.J), the absolute w-score ratio (Figure S3.K) and the spatial prevalence (Figure S3.L) and found this exclusion did not impact our original results. In the same manner we excluded the top percentage of autism individuals with a high Euler index from the analysis (Figure S3M-O) and again found no impact on our original results.

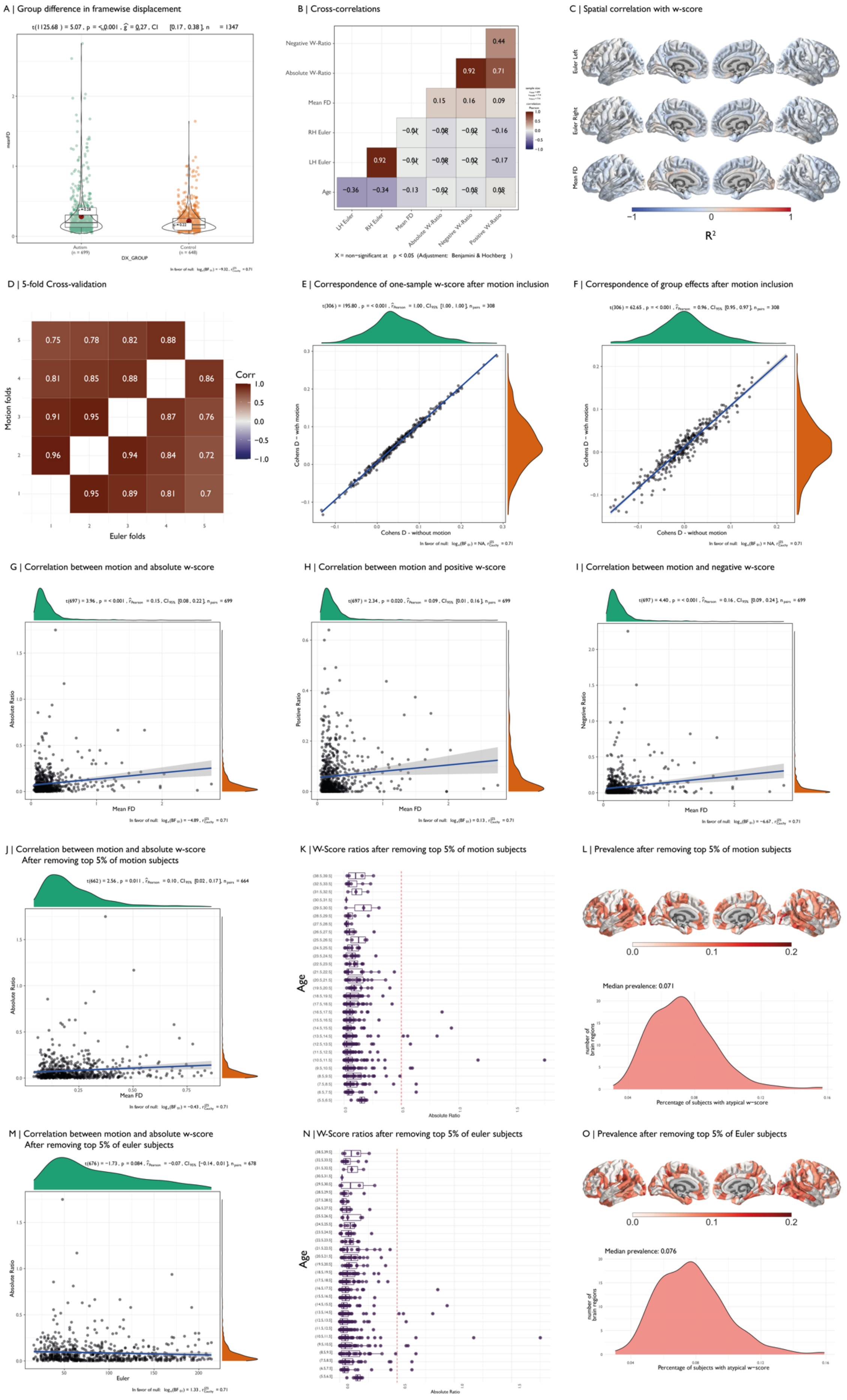

A | Group difference in framewise displacement
B | Cross-correlations
C | Spatial correlation with w-score
D | 5-fold Cross-validation
E | Correspondence of one-sample w-score after motion inclusion
F | Correspondence of group effects after motion inclusion
G | Correlation between motion and absolute w-score
H | Correlation between motion and positive w-score
I | Correlation between motion and negative w-score
J | Correlation between motion and absolute w-score
After removing top 5% of motion subjects
K | W-Score ratios after removing top 5% of motion subjects
L | Prevalence after removing top 5% of motion subjects
M | Correlation between motion and absolute w-score
After removing top 5% of euler subjects
N | W-Score ratios after removing top 5% of euler subjects
O | Prevalence after removing top 5% of Euler subjects
Median prevalence: 0.071
Median prevalence: 0.076
Percentage of subjects with atypical w-score
Motion folds
Euler folds
Corr
Mean FD
Absolute Ratio
Age
R²
X = non-significant at p < 0.05 (Adjustment: Benjamini & Hochberg)

Supplemental Figure 3: Sensitivity analyses

Panel A shows the case-control difference in mean framewise displacement, indicating a significantly higher mean framewise displacement in the autism group t(1125.68)= 5.07, p < .001. Panel B shows the Pearson r correlations between age and the confound variables included in our models. Correlations not passing FDR correction of p <.05 are marked with a cross. Panel C shows the spatial correlation of each ROI with the three included confound regressors in our model, all three show small correlations ranging from r = -.18 to r = .14 and were thus included in all subsequent analyses. Panel D shows the spatial correlation between Cohen's D maps from analyses where subject with either high motion (upper triangle) or high Euler indices (lower triangle) were iteratively excluded. The fold refers to the cohorts of exclusion ranging from 1 = 5% exclusion to 5 = 25% excluded. Panel E shows the correspondence in the one-sample model with and without motion included. Models show highly similar spatial topology (r = 1.00, p < .001, BF = Inf). Panel F shows the significant spatial correspondence for the between group linear mixed effects model (r = 0.96, p < .001, BF = Inf). Panel G shows the small relation between the absolute w-score and mean framewise displacement (r = 0.15, p < .001, BF -4.89). Panels H and J shows the same for the positive (r = 0.09, p < .05, BF = 0.13) and negative (r = 0.16, p < .001, BF = -6.67) ratio's respectively. Panel J shows the residual correlation between motion and the absolute ration after excluding the top 5% of motion subjects from the ASD sample (r = 0.10, p <.05, BF = - 0.43). Panel K shows the absolute w-score ratio with the dotted line indicating the cut-off of 0.5 after excluding the top 5% of motion individuals from the ASD sample. Panel L replicates the main figure 3 of this thresholded sample. Panel M shows the residual correlation between the absolute w-score ratio and the Euler index after thresholding the ASD sample at 5% of Euler scores (r = -0.07, p = .08, BF = 1.33). Panel N shows the absolute w-score in the thresholded sample with the dotted line indicating the 0.5 cut-off. Panel O replicated main figure 3 in this thresholded sample.

Bootstrapping

To assess the reliability of this w-score we bootstrapped the normative sample (1000 bootstraps, with replacement) and computed 1000 bootstrapped w-scores for each individual and each brain region. To subsequently quantify the reliability of the w-score we computed an FDR corrected analogous p-value for each subject by computing the absolute position of the real w-score in the distribution of bootstrapped w-scores. The rationale being that if a real w-score would be in the top 5% of the bootstrapped distribution it would likely not be a reliable score (e.g. the score would be influenced by only a small subset of the normative data). The median number of brain regions per subject with a significant p-value was 1 (out of 308), indicating that the w-score provides a robust measure of atypicality.

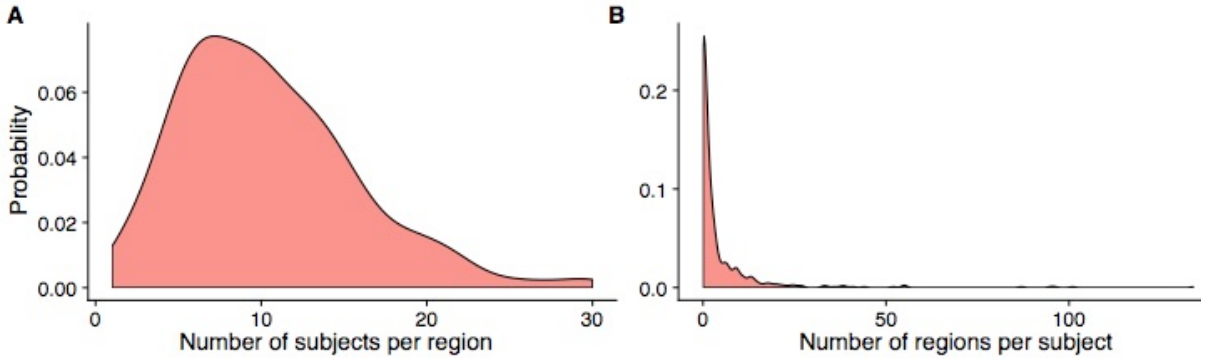

Supplemental Figure 4: bootstrap validation

Panel A shows the probability density distribution for the number of subjects likely to have an unreliable w-score in a given region. For a given region there is a median of 10 subjects (out of 699) for which the w-score is not reliable (e.g. has a p-value <0.05). Panel B shows the probability density distribution for the number of regions likely to have an unreliable w-score in a given subject. The median number of 'unreliable' brain regions per subject is 1.

Comparison to centile modelling of normative deviation

In order to assess the sensitivity of our approach in the present data we implemented the aforementioned bootstrapping procedure to identify robustness of outlier detection. In addition, we also conducted a centiles estimation that is relatively standard in for example epidemiology (Visser et al., 2009), similar to quantile rank maps (Chen et al., 2015) and arguably less sensitive to small sample uncertainty. Both approaches showed high significant correlation in determining whole-brain w-score ratios (r=0.87, p=4e-119 and r=0.66, p=5.7e-39 for ABIDE I and ABIDE II respectively).

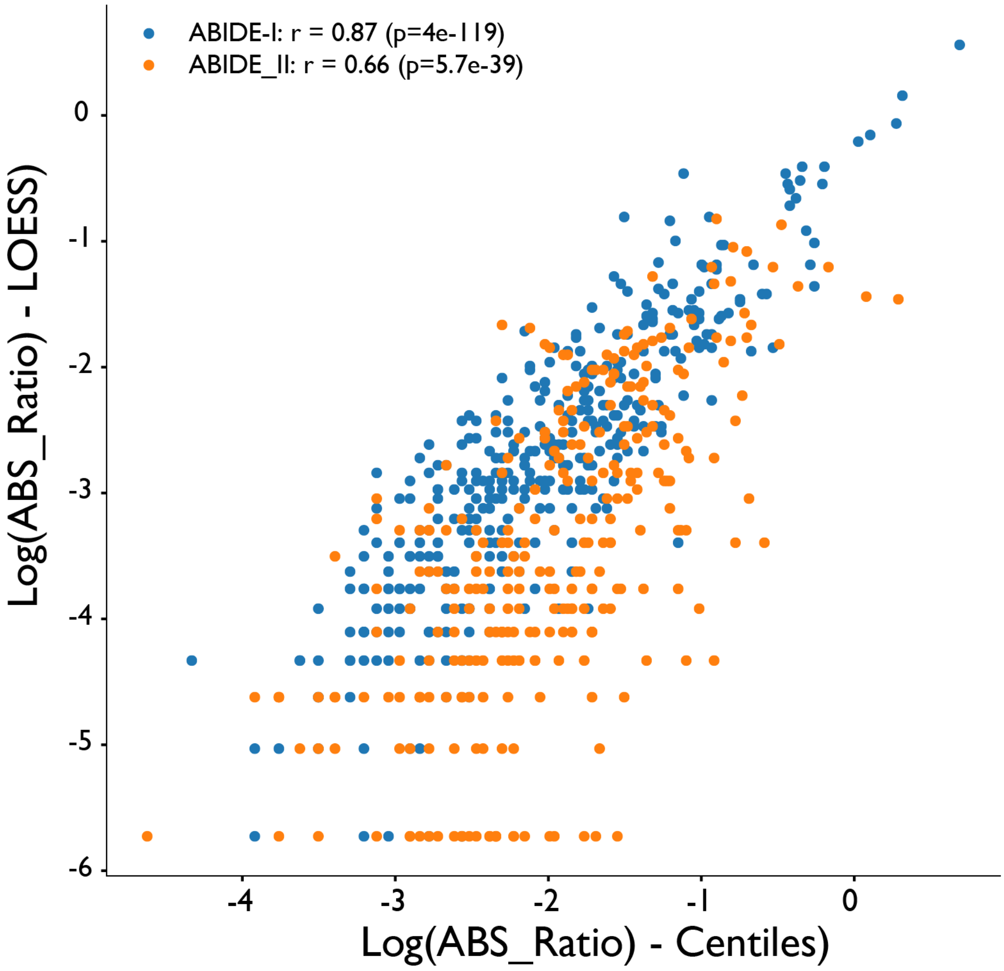

Supplemental Figure 5: Centile vs LOESS regression

Scatterplot of the absolute ration of atypical regions in both ABIDE I and ABIDE II as computed using LOESS regression and centiles estimation.

Individual summary ratios

To isolate subsets of individuals with significant age-related CT deviance, we used a cut-off score of 2 standard deviations (i.e. w >= 2 or w<=2). This cut-off allows us to isolate specific ASD patients with markedly abnormal CT relative to age-norms for each individual brain region. We then calculated sample prevalence (percentage of all ASD patients with atypical w-scores), in order to describe how frequent such individuals are in the ASD population and for each brain region individually. A sample prevalence map can then be computed to show the

frequency of these patients across each brain region. We also wanted to assess how many patients have markedly atypical w-scores (beyond 2SD) across a majority of brain regions. This was achieved by computing an individual global w-score ratio as follows:

$$gW = \frac{\sum|(w)| > 2}{\sum|(w)| < 2}$$

We also computed global w-score ratios for positive and negative w regions separately.

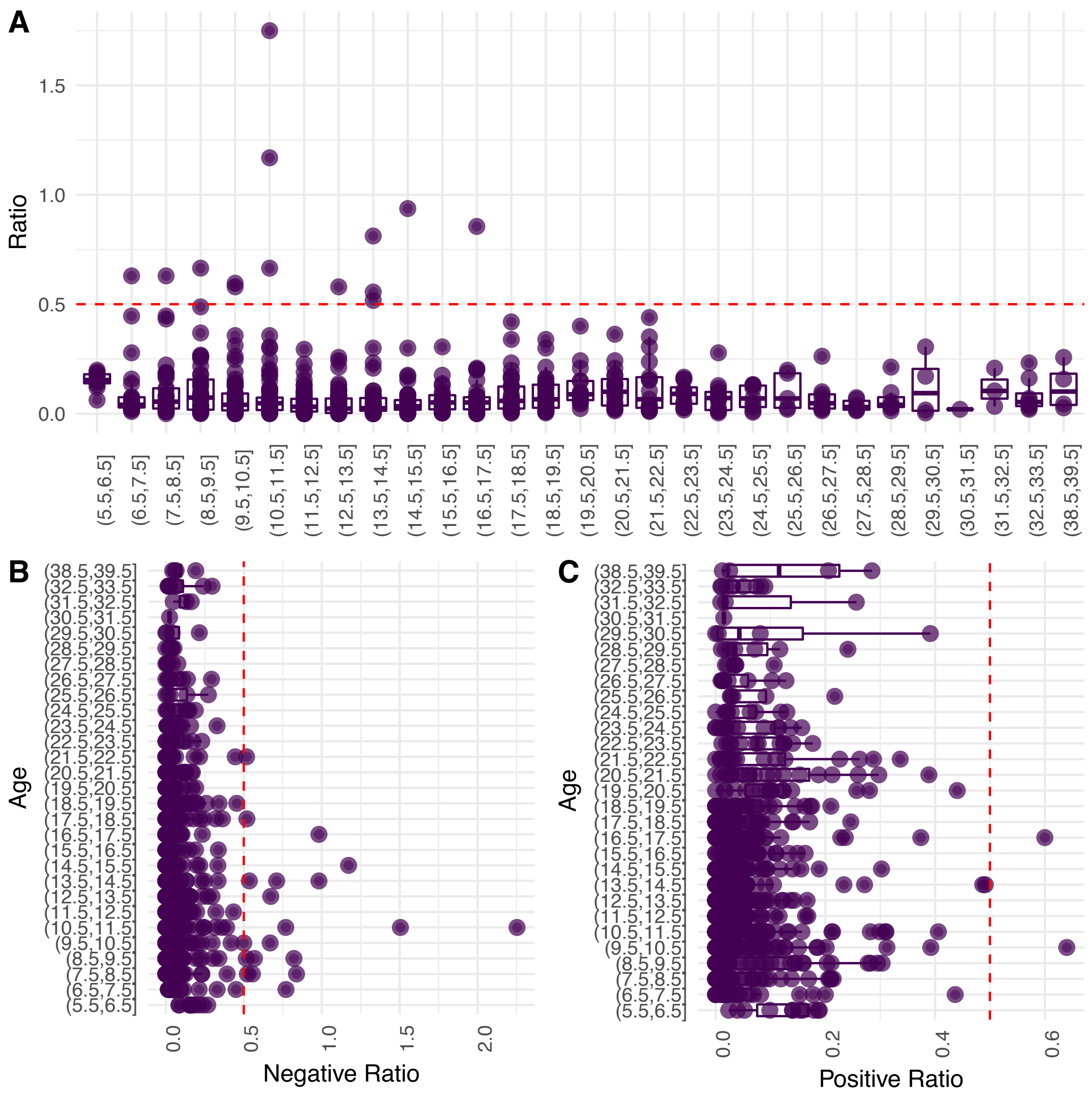

Supplemental Figure 6: Global individual W-Score ratios

Panel A shows the distribution of absolute global ratio scores for each age-bin. There is a total of 14 subjects for which the ratio score exceeds 0.5 meaning they have more atypical than typical regions. Panels B and C show the same but stratified for positive and negative outliers.

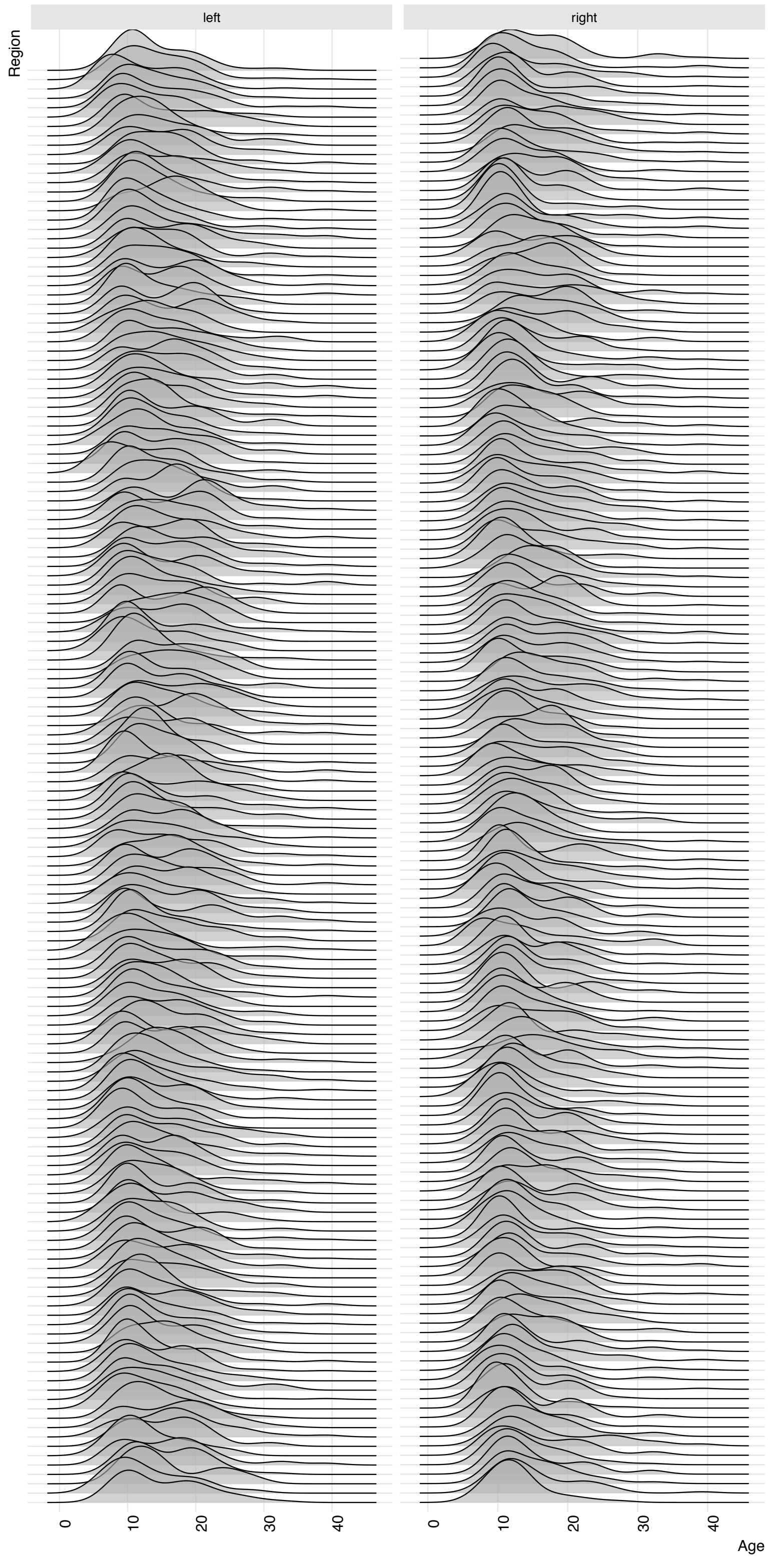

Supplemental Figure 7: Outlier age distribution per brain region

Probability density plots of the age of all outliers for each brain region. Left and right refer to left and right hemisphere.

Age-related CT deviance relationships with SRS and ADOS

An additional advantage of the use of normative modelling over the traditional case-control modelling is that we can use the individualized deviation as a novel metric for finding associations with phenotypic features. Here we used w-scores to compute Spearman correlations for the most commonly shared phenotypic features in the ABIDE dataset: ADOS, SRS, SCQ, AQ, FIQ and Age. After correcting for multiple comparisons across phenotype and region (6 phenotypic measures * 308 regions = 1848 tests) we identified a number of brain regions that survive multiple comparison corrections for the SRS and ADOS scores (supplementary figure S8). SRS is associated with w-scores primarily in areas of lateral frontal and parietal cortex, while ADOS is associated with w-scores primarily in lateral and inferior temporal cortex. Notably, these regions are largely different from regions that appear to show on-average differentiation in case-control and w-score analyses.

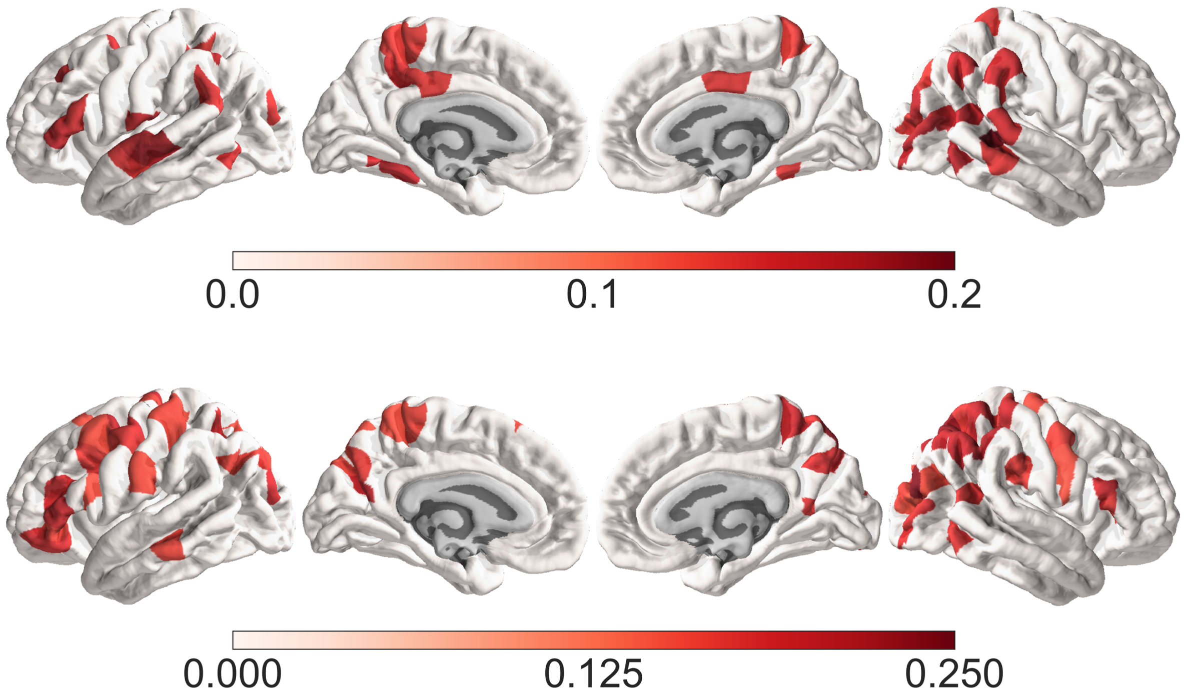

Supplemental Figure 8: Phenotype – W-Score correlations

Spearman correlations between ADOS and w-score in the top panel. The lower panel shows the same for the SRS.

Surface area, LGI and Volume

We applied the same approach to quantify outlier contribution and assess overall variance contribution in surface area, LGI and cortical volume. All three metrics consistently showed strong influence of sex and scanner site as important covariates (S8). In addition, cortical volume also showed a strong influence of age. For all three measures all canonical case-control differences, derived from standard LME modelling, disappear when region-wise outliers were removed (S9-S11). This strongly suggest that the majority of broad case-control differences were driven by a subgroup of individual outliers.

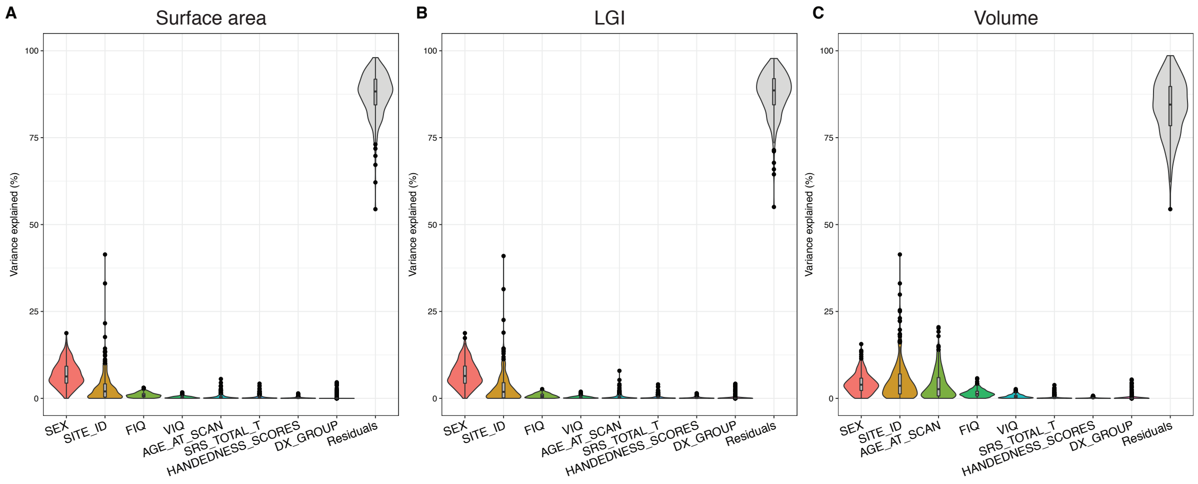

Supplemental Figure 9: Variance contribution across measures

Gender and scanning site are the dominant sources of covariance

Volume

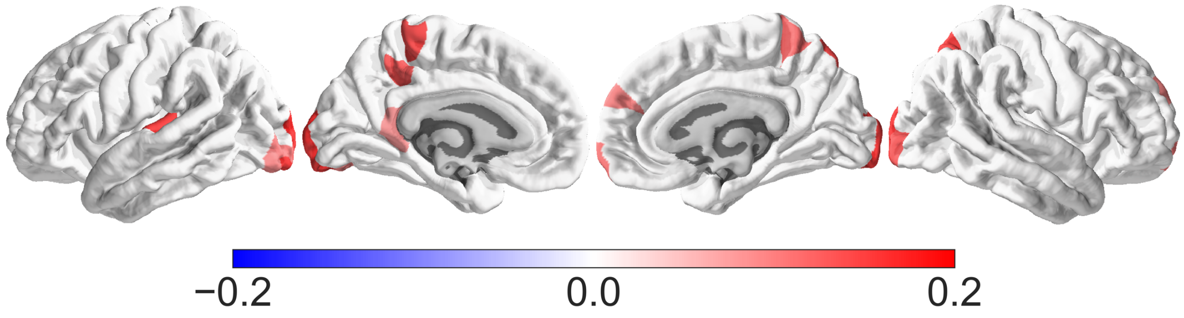

Supplemental Figure 10: Canonical case-control comparisons for cortical volume

The top panel shows the canonical case-control output. No regions passed FDR when region-wise outliers were removed nor on the one sample w-score test.

LGI

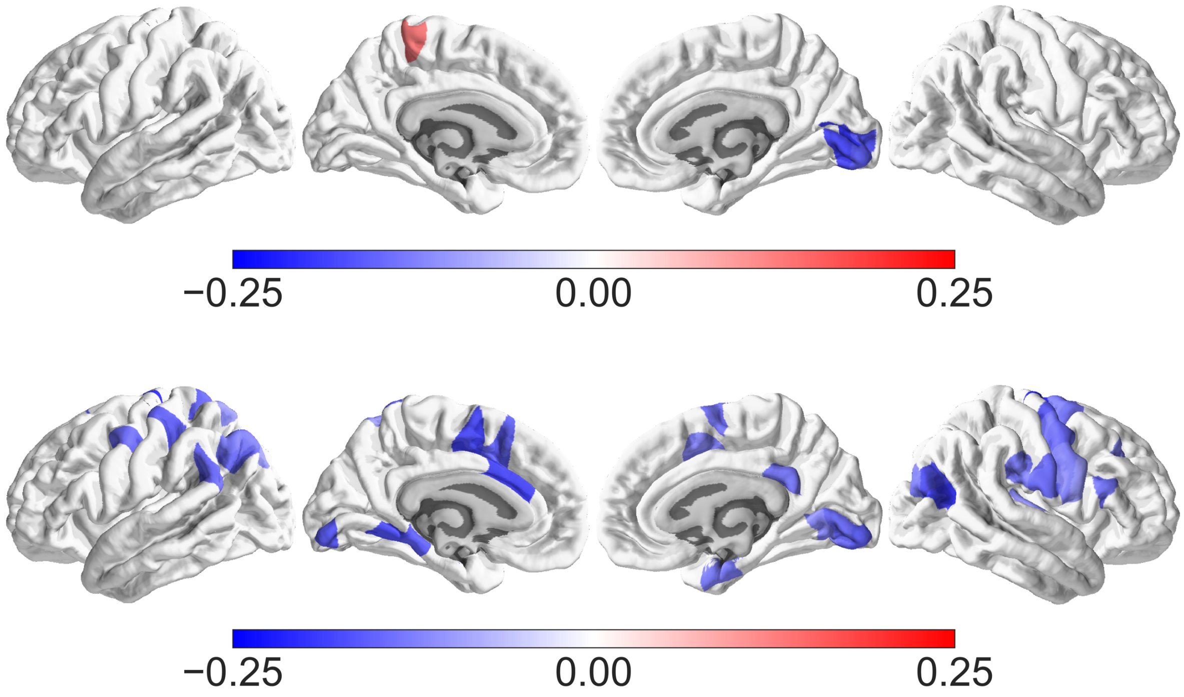

Supplemental Figure 11: Canonical case-control comparisons for local gyrification

The top panel shows the canonical case-control output. No regions pass FDR when region-wise outliers are removed however in the w-score one sample test (lower panel) there are a number of regions that show significantly smaller LGI in ASD.

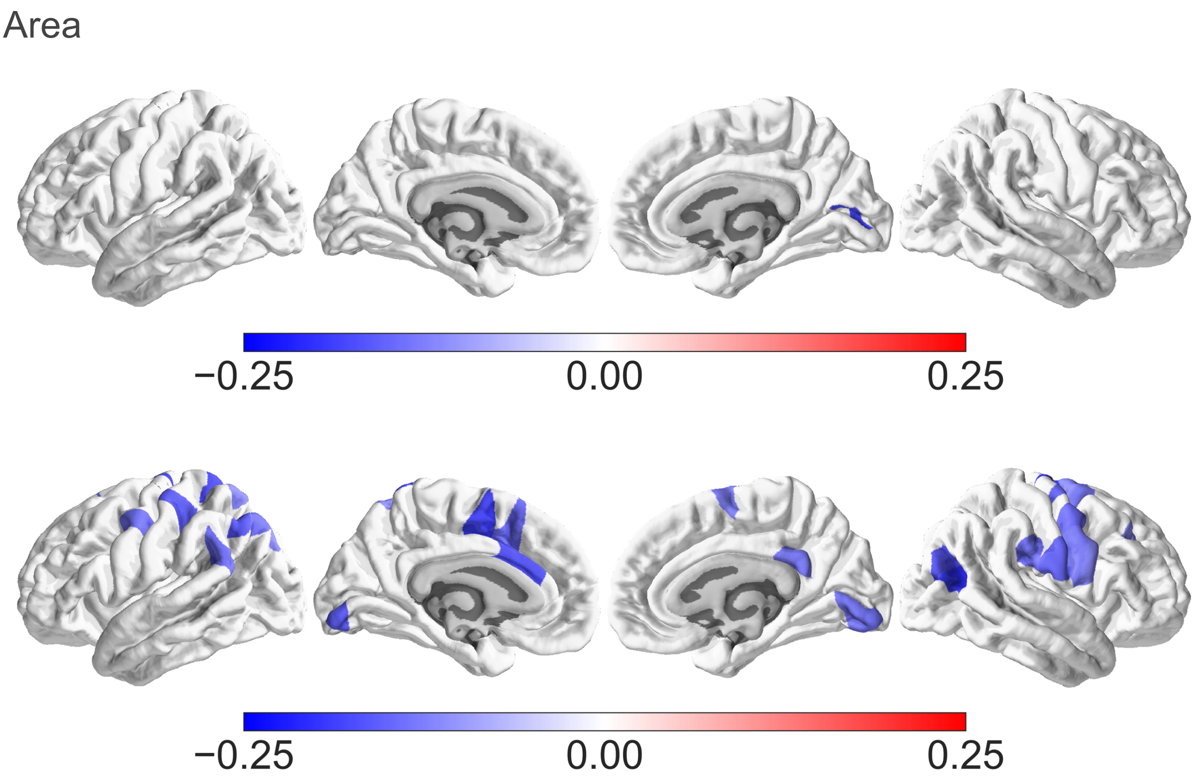

Supplemental Figure 12: Canonical case-control comparisons for surface area

The top panel shows the canonical case-control output. No regions pass FDR when region-wise outliers are removed however in the w-score one sample test (lower panel) there are a number of regions that show significantly smaller surface area in ASD.

Multivariate clustering

Using t-Distributed Stochastic Neighbour Embedding (tSNE) was used to construct a distance matrix from the 308 ROI features. K-medoid clustering on this distance matrix was used to cluster subjects into maximally independent groups using the optimum average silhouette width (Hennig and Liao, 2013) to determine the optimal number of cluster. This identified an optimal number of two clusters. Finally, we re-ran k-medoid clustering with 2 clusters and explored the overlap these clusters gave with diagnosis by visualizing the clustering onto the 2-dimensional embedded space obtained from tSNE.

Despite the limited main diagnosis effect on CT over the majority of brain regions and the fact that only a small subset of individuals appears to contribute to this difference, it may still be possible that the multivariate patterning in CT may capture some diagnostic effect. Thus, we

performed exploratory clustering analysis to determine if raw CT values across the whole brain could be used to delineate the ASD group from the TD group. In addition, we reasoned a data-driven clustering approach might also reveal subgroups within each group (Lombardo et al., 2016). Results from clustering the neighbour embedded raw CT scores are shown in Figure 6. As can be observed in panel B, the within-group heterogeneity is entirely captured by normative heterogeneity and the overall density plots for both groups are close to identical. The pattern we find when projecting the whole brain raw cortical thickness into a 2-dimensional embedding most closely resembles the 3rd scenario outlined by Marquand and colleagues (Marquand et al., 2016), whereby disease related variation is nested with the normal variation. Our results show that, when it comes to whole-brain cortical thickness, the condition related variation is entirely nested within the neurotypical variation. Obviously, the present clustering and embedding approaches only provide one way of clustering or segregating case-control variation in cortical thickness. Other multivariate approaches that took into account a multitude of variables did reveal that multivariate clustering has the potential to identify subgroups (Hong et al., 2017). Additionally, other measures than CT might provide a different picture. In is interesting however to note that both dimensions were correlated with age. No correlations were observed with any of the other common phenotypic measures. Thus, this 2-dimensional embedding likely captures the variability in cortical thickness expansion and thinning over the course of development, but is not sensitive enough to pick up potential alterations in the overall trajectory of this process between groups.

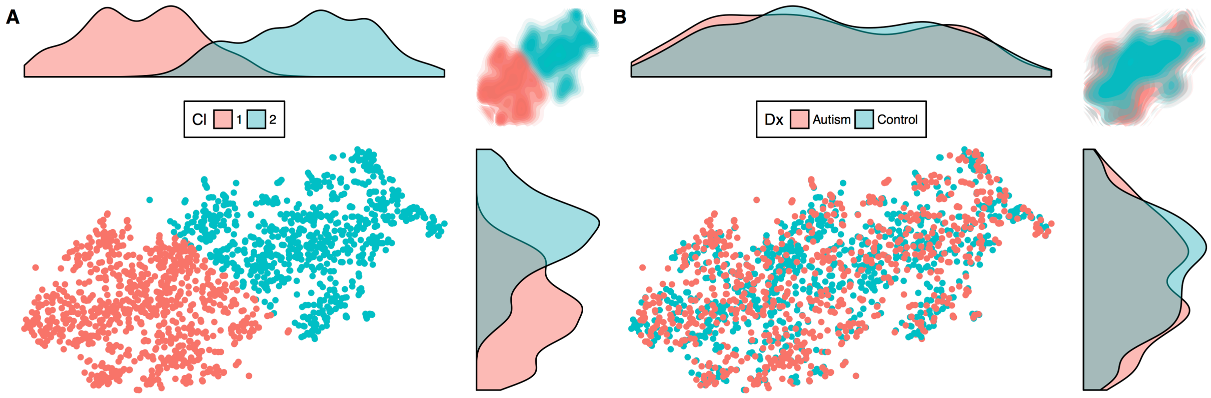

Supplemental Figure 13: tSNE Clustering

Panel A shows the results from k-medoid clustering of the 2D embedding of the raw CT values as achieved by tSNE. K-medoid clustering clearly identifies 2 clusters. However, as Panel B shows, these clusters did not provide any meaningful distinction on diagnosis.

References

Chen H, Kelly C, Xavier Castellanos F, He Y, Zuo X-N, Reiss PT. 2015. Quantile rank maps: A new tool for understanding individual brain development. Neuroimage 111:454–463.

Hennig, C., Liao, T.F., 2013. How to find an appropriate clustering for mixed-type variables with application to socio-economic stratification. J. R. Stat. Soc. Ser. C (Applied Stat. 62, 309–369. https://doi.org/10.1111/j.1467-9876.2012.01066.x

Hong, S., Valk, L., Martino, A. Di, Milham, M.P., Bernhardt, B.C., 2017. Multidimensional Neuroanatomical Subtyping of Autism Spectrum Disorder 1–11. https://doi.org/10.1093/cercor/bhx229

Lombardo, M. V, Lai, M.-C., Auyeung, B., Holt, R.J., Allison, C., Smith, P., Chakrabarti, B., Ruigrok, A.N. V, Suckling, J., Bullmore, E.T., MRC AIMS Consortium, Ecker, C., Craig, M.C., Murphy, D.G.M., Happé, F., Baron-Cohen, S., 2016. Unsupervised data-driven stratification of mentalizing heterogeneity in autism. Sci. Rep. 6, 35333. https://doi.org/10.1038/srep35333

Marquand, A.F., Rezek, I., Buitelaar, J.K., Beckmann, C.F., 2016. Understanding Heterogeneity in Clinical Cohorts Using Normative Models: Beyond Case-Control Studies. Biol. Psychiatry 80, 552–561. https://doi.org/10.1016/j.biopsych.2015.12.023

Rosen, A.F.G., Roalf, D.R., Ruparel, K., Blake, J., Seelaus, K., Villa, L.P., Ciric, R., Cook, P.A., Davatzikos, C., Elliott, M.A., Garcia de La Garza, A., Gennatas, E.D., Quarmley, M., Schmitt, J.E., Shinohara, R.T., Tisdall, M.D., Craddock, R.C., Gur, R.E., Gur, R.C., Satterthwaite, T.D., 2018. Quantitative assessment of structural image quality. Neuroimage 169, 407–418. https://doi.org/10.1016/j.neuroimage.2017.12.059

Visser GHA, Eilers PHC, Elferink-Stinkens PM, Merkus HMWM, Wit JM. 2009. New Dutch reference curves for birthweight by gestational age. Early Hum Dev 85:737–744.
