# Supplementary material for: A normative modelling approach reveals age-atypical cortical thickness in a subgroup of males with autism spectrum disorder
Source: Commun Biol. 2020 Sep 4;3:486. doi: 10.1038/s42003-020-01212-9 (PMC7474067; doi:10.1038/s42003-020-01212-9)
Supplement: Supplementary file 2 — Reporting Summary [file 42003_2020_1212_MOESM2_ESM.pdf]

nature research

Corresponding author(s): Richard A.I. Bethlehem

Last updated by author(s): Jul 22, 2020

Reporting Summary

Nature Research wishes to improve the reproducibility of the work that we publish. This form provides structure for consistency and transparency in reporting. For further information on Nature Research policies, see our Editorial Policies and the Editorial Policy Checklist.

Statistics

For all statistical analyses, confirm that the following items are present in the figure legend, table legend, main text, or Methods section.

n/a	Confirmed	
☐	☒	The exact sample size (*n*) for each experimental group/condition, given as a discrete number and unit of measurement
☐	☒	A statement on whether measurements were taken from distinct samples or whether the same sample was measured repeatedly
☐	☒	The statistical test(s) used AND whether they are one- or two-sided<br>*Only common tests should be described solely by name; describe more complex techniques in the Methods section.*
☐	☒	A description of all covariates tested
☐	☒	A description of any assumptions or corrections, such as tests of normality and adjustment for multiple comparisons
☐	☒	A full description of the statistical parameters including central tendency (e.g. means) or other basic estimates (e.g. regression coefficient) AND variation (e.g. standard deviation) or associated estimates of uncertainty (e.g. confidence intervals)
☐	☒	For null hypothesis testing, the test statistic (e.g. *F*, *t*, *r*) with confidence intervals, effect sizes, degrees of freedom and *P* value noted<br>*Give P values as exact values whenever suitable.*
☐	☒	For Bayesian analysis, information on the choice of priors and Markov chain Monte Carlo settings
☐	☒	For hierarchical and complex designs, identification of the appropriate level for tests and full reporting of outcomes
☐	☒	Estimates of effect sizes (e.g. Cohen's *d*, Pearson's *r*), indicating how they were calculated

Our web collection on statistics for biologists contains articles on many of the points above.

Software and code

Policy information about availability of computer code

Data collection	All code and data is available from GitHub: https://github.com/rb643/Normative_modeling, all data used in the study is publicly available from the ABIDE consortium
Data analysis	All code and data is available from GitHub: https://github.com/rb643/Normative_modeling, all data used in the study is publicly available from the ABIDE consortium

For manuscripts utilizing custom algorithms or software that are central to the research but not yet described in published literature, software must be made available to editors and reviewers. We strongly encourage code deposition in a community repository (e.g. GitHub). See the Nature Research guidelines for submitting code & software for further information.

Data

Policy information about availability of data

All manuscripts must include a data availability statement. This statement should provide the following information, where applicable:

- Accession codes, unique identifiers, or web links for publicly available datasets
- A list of figures that have associated raw data
- A description of any restrictions on data availability

All code and data is available from GitHub: https://github.com/rb643/Normative_modeling, all data used in the study is publicly available from the ABIDE consortium

Field-specific reporting

Please select the one below that is the best fit for your research. If you are not sure, read the appropriate sections before making your selection.

☒ Life sciences ☐ Behavioural & social sciences ☐ Ecological, evolutionary & environmental sciences

For a reference copy of the document with all sections, see nature.com/documents/nr-reporting-summary-flat.pdf

Life sciences study design

All studies must disclose on these points even when the disclosure is negative.

Sample size

Because of power limitations in past work with small samples, we conducted an a priori statistical power analysis indicating that a minimum case-control effect size of d = 0.1752 could be detected at this sample size with 80% power at a conservative alpha set to 0.005 (Benjamin et al. 2018). For correlational analyses looking at brain-behaviour associations, we examined a subset of patients with the data from the SRS (N autism_male = 421) and ADOS total scores (N autism_male = 505). With the same power and alpha levels the minimum effect for SRS is r = 0.1765 and r = 0.1651 for the ADOS.

Data exclusions

"The Euler number is a quantitative proxy index of segmentation quality and has shown high overlap with manual quality control labelling (Rosen et al 2016). The index counts the number of times the freesurfer has had to interpolate surface gaps during the reconstruction to ensure a continuous outcome surface. As such the index is effectively a measure for the reliability of the surface reconstruction and the resulting CT estimates. In the full sample we found a small but significant difference in both hemispheres (Figure S2) with the autism group having overall slightly worse scan quality (d = 0.176 and d = 0.187 for left and right hemisphere respectively). Therefore, we chose to exclude the top 10% of subjects with an extreme Euler index (corresponding to a Euler index of approximately 300) and reran the Matchit genetic matching algorithm to check for matched samples. To further ensure adequate control for scan quality we included the index itself as a confound variable in all models."

"Unfortunately, despite a significant female sub-group, the age-wise binning greatly reduced the number of bins with enough data-points in the female group. Given the reduced sample size in the female group and the known interaction between autism and biological sex, as well as the known sex differences in developmental trajectories, we conducted normative modelling on the male group only (Figure 2A)."

In addition, we performed sensitivity analyses by systematically excluding high motion and high Euler individuals from the analyses. These are detailed in a separate section on sensitivity analysis in the results as well as in the supplementary materials.

Replication

Unfortunately we do not have access to a comparable dataset of large enough sample size and age range for replication. Instead we performed extensive permutation analysis on our approach to ensure reliability of derived scores.

"To assess the reliability of the normative w-score we permuted the normative sample (1000 bootstraps, with replacement) and computed 1000 permuted w-scores for each individual and each brain region. To subsequently quantify the reliability of the w-score we computed an FDR corrected analogous p-value for each subject by computing the absolute position of the real w-score in the distribution of permuted w-scores. The rationale being that if a real w-score would be in the top 5% of the bootstrapped distribution it would likely not be a reliable score (e.g. the score would be influenced by only a small subset of the normative data). The median number of brain regions per subject with a significant p-value in the normative sample was 1 (out of 308), indicating that the normative sample is topology robust and that the w-score is a robust reflection of atypicality. More details on the bootstrapping procedure are provided in the supplementary material (SI: Boostrapping and SI figure S4)."

Randomization

Normative modeling aims to treat every clinical individual as an individual data in reference to a population norm, thus no group randomization was conducted. As noted above however we did perform extensive permutation tests (shuffling the individuals ID label) to assess the stability and reliability of the derived scores.

Blinding

No blinding was necessary in the present analysis

Reporting for specific materials, systems and methods

We require information from authors about some types of materials, experimental systems and methods used in many studies. Here, indicate whether each material, system or method listed is relevant to your study. If you are not sure if a list item applies to your research, read the appropriate section before selecting a response.

Materials & experimental systems

n/a	Involved in the study
☒	☐ Antibodies
☒	☐ Eukaryotic cell lines
☒	☐ Palaeontology and archaeology
☒	☐ Animals and other organisms
☐	☒ Human research participants
☒	☐ Clinical data
☒	☐ Dual use research of concern

Methods

n/a	Involved in the study
☒	☐ ChIP-seq
☒	☐ Flow cytometry
☐	☒ MRI-based neuroimaging

Human research participants

Policy information about studies involving human research participants

Population characteristics	All sample characteristics are provided in tables 1-3. An assessment of potentially relevant covariates is included in the methods section and summarized in Figure 5.
Recruitment	Existing publicly available data from ABIDE was used.
Ethics oversight	Existing publicly available data from ABIDE was used. ABIDE is an aggregated ananomized dataset where research institutes can deposit their data openly. Participants included provided written and informed consent at the institute where they participated and all these centers had their own ethical oversight in place. More information on sites can be found on: http://fcon_1000.projects.nitrc.org/indi/abide/

Note that full information on the approval of the study protocol must also be provided in the manuscript.

Magnetic resonance imaging

Experimental design

Design type	Normative modeling of existing brain anotomy
Design specifications	N/A
Behavioral performance measures	N/A

Acquisition

Imaging type(s)	Structural T1 weighted imaging
Field strength	3T
Sequence & imaging parameters	Mainly MPRAGE, parameters for each site can be found on: http://fcon_1000.projects.nitrc.org/indi/abide/
Area of acquisition	Whole-brain

Diffusion MRI ☐ Used ☒ Not used

Preprocessing

Preprocessing software	Freesurfer v5.3
Normalization	Standard intensity normalization included in freesurfer. No MNI warping done.
Normalization template	N/A
Noise and artifact removal	N/A
Volume censoring	N/A

Statistical modeling & inference

Model type and settings	Regional linear mixed effect models
Effect(s) tested	*Define precise effect in terms of the task or stimulus conditions instead of psychological concepts and indicate whether ANOVA or factorial designs were used.*

Specify type of analysis: ☐ Whole brain ☐ ROI-based ☐ Both

Statistic type for inference (See Eklund et al. 2016)	Effect of autism diagnosis
Correction	FDR correction across brain regions/parcels and Monte-Carlo permutations (1000) for stability estimation.

Models & analysis

n/a	Involved in the study
☒	☐ Functional and/or effective connectivity
☒	☐ Graph analysis
☒	☐ Multivariate modeling or predictive analysis
